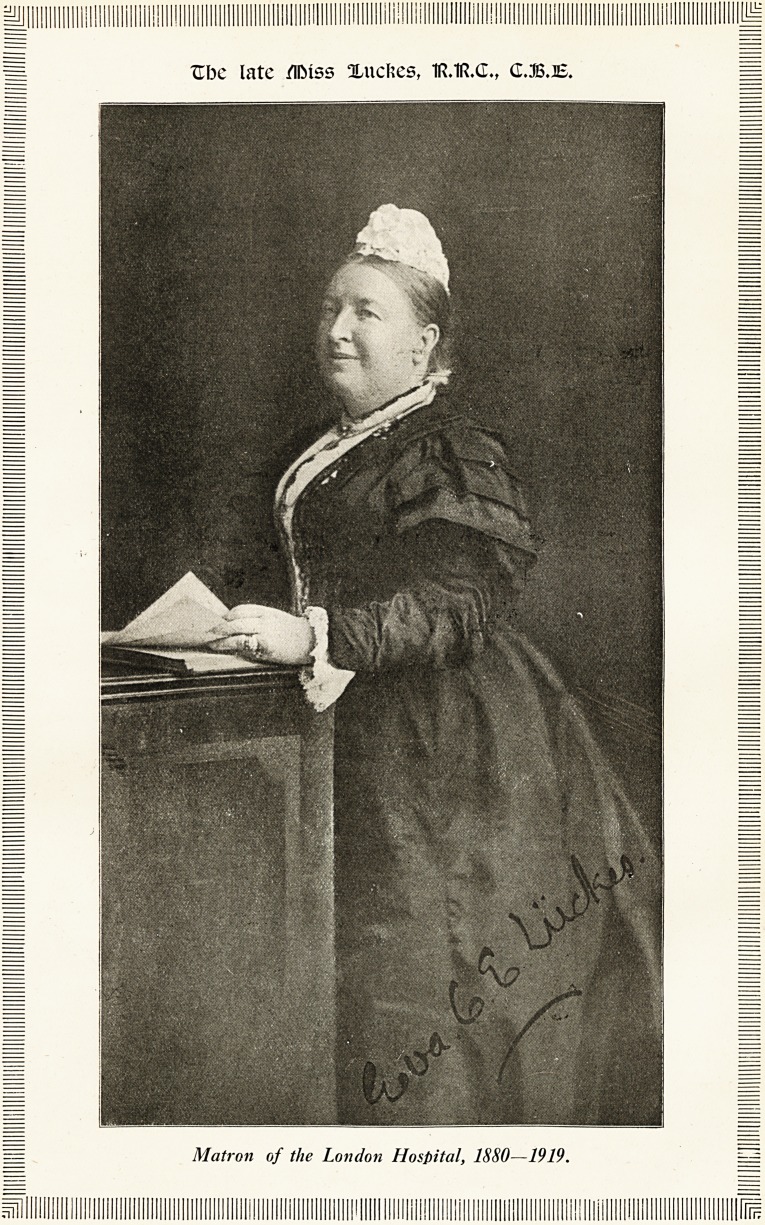# Miss Eva Luckes—A Great Woman

**Published:** 1919-02-22

**Authors:** 


*Matron of the London Hospital, 1880—1919.*

### MISS EVA LUCKES—A GREAT WOMAN.

### AN APPRECIATION FROM THE LONDON HOSPITAL.

Miss Eva Luckes, R.R.C., Lady of Grace of the Order of St. John of Jerusalem, Commander of the Order of the British Empire, Matron of the London Hospital for thirty-nine years, passed peacefully to her rest on Sunday afternoon, the 16th inst., after a very long illness, and after cremation was buried three days later at Golder's Green. The memorial service will be at the hospital church, St. Philip's, Stepney, on Saturday, February 22. Her death marks the definite close of the stirring period in nursing history which began when Florence Nightingale dedicated the nation's gift to the foundation of a training school for nurses. Miss Luckes, whose name points to a Danish ancestor, was trained at the Westminster Hospital in the training-school initiated there after the example of the Nightingale School. Practically her entire life-work was carried on at the London Hospital, where out of nursing chaos her long labours have resulted in building up a training-school for nurses and a private nursing staff unique in numbers and organisation throughout the Empire. Throughout her long career she received many tokens of regard and respect from various members of the Royal Family, and Queen Alexandra, in particular, delighted in her many gifts and her rich and distinctive personality. We append an appreciation of her work and character written by one who, more perhaps than any other, had opportunities of gauging her qualities of mind and heart.

---

What have I found in her? Perhaps what has struck me most has been her instinctive knowledge of what is right—not only wise, but right. For this she would work, and from this she could not be diverted. The second best would never do. She would accept defeat for the moment if she could not get what she thought right, and would then begin working for it again. She knew what she wanted, and felt sure that in time she would get it. She did get it. That determination has made its mark on the hospital, where to-day all her ideas have been carried out.

### Many Reforms Initiated.

For years she advocated the starting of a preliminary training-school which then existed nowhere else. At last such a school, Tredegar House, was opened in 1895. She was never tired of preaching that nurses should be properly housed and fed. There are now four nurses' homes, giving to every nurse a good bedroom to herself, with cheerful sitting-rooms, library, etc. One is called The Eva Luckes' Home, after her. To the food of the nurses Miss Luckes gave her personal attention, seeing the menu every day. Then by degrees she succeeded in giving the nurses longer holidays, increasing the time off every day from one to three hours, in securing days off every fortnight, in arranging long leave. Then she arranged for the study classes to be held during working hours. Gradually the nurses' pay increased and a full-pay pension after eighteen years of work was assured to every nurse.

### The London System.

She believed strongly in the advantage of keeping the nurses sent out to nurse private patients in close touch with the hospital, and in not separating the private staff from the working staff by keeping up a separate establishment for them. Her idea of having the nurses back to the wards between their cases, after sufficient rest, was carried out. Her method of training nurses was her own. After seven weeks at the preliminary training-school and the qualifying examination, the nurses attend lectures and small study classes during their first year. During that year there are two examinations, one on medical and one on surgical nursing, and at the end of it there is a final examination. Then the nurses are free of all examinations, and at the end of the second year, if they deserve it, they are given the London Hospital certificate of a trained nurse. By the agreement signed when they entered they have, however, to serve the hospital for another two years, spending, if they wish it, one of these years, not necessarily the third, in the hospital. Miss Luckes insisted that the best way to fit a woman to be a good and efficient nurse was to give her responsibility directly she had shown herself capable of taking it. Probationers who showed special ability were given an "S" and allowed to do staff-nurse duty under the careful supervision of the sisters before they gained their certificates. She was a very good judge of character as to who should be given responsibility.

### Special Talents and Characteristics.

She had a wonderful capacity for work. She radiated cheerfulness. No detail, either personal or official, was too small to receive her fullest attention, and no matter how busy she was, any one talking to her felt for the time being she was absolutely concentrating her attention on the matter in hand. She gave her unbiased opinion freely and fearlessly. Her absolute and obvious sincerity created the utmost confidence in her judgment. Matron's absolute unselfishness, self-control, and cheerful devotion to work were ever a great and shining example. She had a splendid courage, and a hidden inexhaustible source of strength which she freely poured out. She had the same wonderful memory for faces and people as Queen Victoria. Once she had seen a nurse, once let her know something about the nurse's circumstances, and it seemed impossible for her to forget. Her grasp and quick understanding of details, together with her extraordinary memory, made her a marvellous head of a great training school.

### Influence over her Nurses.

She was indeed a mother in the highest sense of the word, besides being a great teacher and leader. She won the confidence of the shyest and most nervous candidate at the first interview by her great charm of manner, her kindness, gentleness, and quick and ready sympathy. She made a nurse feel at once that for the calling of nursing only the best was worth while. She had the most intense desire to be just and fair, and she always leaned to mercy except when the delinquent had shown any neglect of patients, any want of sympathy, or any frivolity tending to lower her standard of proper behaviour for a nurse. She had a very tender side for anything done through thoughtlessness or even carelessness, provided only that the "nursing instincts" were there. Miss Luckes' lectures to the nurses and her talks to the sisters were much looked forward to. The nurses on the private staff were encouraged to write to her, and received a letter from her nearly every week. In this way she kept in close personal touch with all of them, and was able to help and advise them in many of their troubles and difficulties. They felt they had a strong friend to lean on, and she had the joy of seeing them respond.

### Intimacy with Florence Nightingale.

Miss Nightingale was a very dear friend of hers and consulted her on many nursing and hospital matters. Miss Nightingale constantly wrote to her and was visited by her up to within the last few years of her life. Kinglake wrote of Florence Nightingale: "She founded a gracious dynasty that still reigns supreme in the wards where sufferers lie." The same may be said of Eva Luckes, to Miss Nightingale a worthy successor.

## Figures and Tables

**Figure f1:**